# Lifestyle Modification Using a Wearable Biometric Ring and Guided Feedback Improve Sleep and Exercise Behaviors: A 12-Month Randomized, Placebo-Controlled Study

**DOI:** 10.3389/fphys.2021.777874

**Published:** 2021-11-25

**Authors:** Jonathan D. Browne, David M. Boland, Jaxon T. Baum, Kayla Ikemiya, Quincy Harris, Marin Phillips, Eric V. Neufeld, David Gomez, Phillip Goldman, Brett A. Dolezal

**Affiliations:** ^1^Airway & Exercise Physiology Research Laboratory, David Geffen School of Medicine, University of California Los Angeles, Los Angeles, CA, United States; ^2^School of Medicine, California University of Science and Medicine, Colton, CA, United States; ^3^Army-Baylor University Doctoral Program in Physical Therapy, San Antonio, TX, United States; ^4^School of Medicine, Texas Tech University Health Sciences Center, Lubbock, TX, United States; ^5^Donald and Barbara Zucker School of Medicine at Hofstra/Northwell, Hofstra University, Hempstead, NY, United States; ^6^College of Arts and Sciences, University of Colorado Boulder, Boulder, CO, United States

**Keywords:** wearable, biometric ring, fitness, recovery, step count, heart rate variability, sleep onset latency, VO_2_max

## Abstract

**Purpose:** Wearable biometric monitoring devices (WBMD) show promise as a cutting edge means to improve health and prevent disease through increasing accountability. By regularly providing real-time quantitative data regarding activity, sleep quality, and recovery, users may become more aware of the impact that their lifestyle has on their health. The purpose of this study was to examine the efficacy of a biometric tracking ring on improving sleep quality and increasing physical fitness over a one-year period.

**Methods:** Fifty-six participants received a biometric tracking ring and were placed in one of two groups. One group received a 3-month interactive behavioral modification intervention (INT) that was delivered virtually *via* a smartphone app with guided text message feedback (GTF). The other received a 3-month non-directive wellness education control (CON). After three months, the INT group was divided into a long-term feedback group (LT-GTF) that continued to receive GTF for another nine months or short-term feedback group (ST-GTF) that stopped receiving GTF. Weight, body composition, and VO_2_max were assessed at baseline, 3months, and 12months for all participants and additionally at 6 and 9months for the ST-GTF and LT-GTF groups. To establish baseline measurements, sleep and physical activity data were collected daily over a 30-day period. Daily measurements were also conducted throughout the 12-month duration of the study.

**Results:** Over the first 3months, the INT group had significant (*p*<0.001) improvements in sleep onset latency, daily step count, % time jogging, VO_2_max, body fat percentage, and heart rate variability (rMSSD HRV) compared to the CON group. Over the next 9months, the LT-GTF group continued to improve significantly (*p*<0.001) in sleep onset latency, daily step count, % time jogging, VO_2_max, and rMSSD HRV. The ST-GTF group neither improved nor regressed over the latter 9months except for a small increase in sleep latency.

**Conclusion:** Using a WBMD concomitantly with personalized education, encouragement, and feedback, elicits greater change than using a WBMD alone. Additionally, the improvements achieved from a short duration of personalized coaching are largely maintained with the continued use of a WBMD.

## Introduction

It is now well known that positive lifestyle habits, such as regular exercise and adequate sleep, profoundly affect one’s mental and physical health. A sedentary lifestyle is one of the prominent modifiable contributors of mortality and disease globally. It undoubtedly plays a central role in increasing the risk of developing chronic health conditions, such as cardiovascular disease, hypertension, metabolic syndrome, obesity, cancer, and depression (Richardson et al., 2004; Charansonney and Després, 2010; Teychenne et al., 2010; Tremblay et al., 2010; León-Latre et al., 2014; Mainous et al., 2019), and serves as a major predictor of hospitalization and mortality (Biswas et al., 2015). Despite a widespread understanding of the beneficial aspects of physical activity, roughly half of American adults fail to meet the minimum guidelines for recommended exercise (Piercy et al., 2018; Du et al., 2019; Singh et al., 2020). Similarly, short sleep duration, coupled with deficits in sleep quality or restfulness, predisposes individuals to obesity, mental health conditions, neuroendocrine dysfunctions, and cardiovascular disease (Durmer and Dinges, 2005; Vorona et al., 2005; Mullington et al., 2009; Medic et al., 2017). Recent literature indicates that more than 1 in 3U.S. adults do not regularly get enough restful sleep, defined as at least seven hours per night (Centers for Disease Control and Prevention (CDC), 2009; Gamble et al., 2017). Strategies that assist individuals in adopting and maintaining healthier lifestyles may be an important catalyst in addressing public health concerns worldwide.

A growing body of research indicates that the use of digital health technology, notably “wearables,” facilitates the adoption of healthy behaviors with the potential of playing a salubrious role in disease prevention. Wearable technologies are smart microelectronic devices worn on or close to the body that utilize sensors or micro-controllers to detect, transmit, and analyze physiological parameters in real time. Refined sensory technology is also rapidly advancing within the medical device field (Presti et al., 2020). At times, these devices have demonstrated a profound positive impact on health behaviors, such as exercise, sleep, and more (Dunn et al., 2018; Massaroni et al., 2019). Wearables are uniquely capable in that they leverage the users data to provide motivation and accountability (Bianchi, 2018; Nanda et al., 2019; Düking et al., 2020). During sleep deprivation, wearables can encourage the user to get adequate sleep. By corollary, during extended periods of inactivity, they can remind you to increase your activity level. Commonly, wearables include a screen interface or mobile application to augment intrinsic behavior-regulating strategies, such as goal setting, associations, and self-monitoring. Extrinsic strategies enhanced by digital technology include education, reinforcement, encouragement, and actionable feedback (Bandura, 1991; Asbjørnsen et al., 2019).

While many studies support the efficacy of wearables to positively impact behavior in the short term, there is a dearth of evidence on long-term adherence and adaptation. The use of many devices tends to result in only short-term adoption and temporary changes in motivation and behaviors (Klasnja et al., 2011). In fact, a study by Lee et al. found that one-third of consumers who have owned a wearable device stopped using it within six months (Lee et al., 2016). If wearable devices are to be part of the solution of sustained behavior change, a major challenge indeed, they need to leverage principles from theories of health behavior. Current consumer-grade wearables align well with the behavioral change technique of self-monitoring, but without an action planning and commitment step, they may have less of an impact on actual behavioral change.

Adherence seems to be influenced by the individual’s recognition of long-term benefits, social support, and internal motivation (Kononova et al., 2019). User surveys have shown that low levels of compliance are associated with discomfort, the inconvenience of wearable devices, and a lack of activity specificity that is important for goal setting and reinforcement (McNamara et al., 2016; Faust et al., 2017; Ridgers et al., 2018). Thus, perhaps smaller, lighter, and more inconspicuous wearables that require less frequent charging may mitigate some compliance issues. Additionally, research indicates that integrating wearable devices into more comprehensive, personally tailored behavioral interventions improves compliance and behavior change (Dolezal et al., 2015, 2019).

Behavioral modification interventions can empower positive lifestyle and behavioral changes by improving health education, increasing motivation, and providing actionable goals. The present study was conducted to examine the efficacy of a personalized behavioral modification protocol informed by data from a biometric tracking ring over a one-year period. We hypothesized that the behavioral intervention augmented by wearable technology would have improved adherence and subsequent sleep and exercise outcomes compared to a non-augmented intervention.

## Materials and Methods

### Participants

Volunteers were recruited and enrolled from the University of California, Los Angeles (UCLA) campus and the surrounding Los Angeles area. Inclusion criteria included: (i) men and women aged 18–55years, (ii) little to no exercise in the past 3months (<4x/month), (iii) willingness to wear a ring continuously for the duration of the study (i.e., 12months), and (iv) willingness to refrain from engaging in any new activity outside of the study requirements of walking, jogging, or running. All volunteers completed a pre-participation physical activity readiness questionnaire (PAR-Q) and an exercise history questionnaire. Exclusion criteria included: (i) any significant medical diagnoses, including cardiovascular or pulmonary disease, that may limit the ability to exercise or increase the cardiovascular risk of exercising, and (ii) failing to meet the criteria for low or moderate risk of exercise participation as defined by American College of Sports Medicine Guidelines. The research was a year-long randomized, placebo-controlled study using healthy participants enrolled from October 2018 to November 2019. All participants provided written informed consent prior to study onset. The study was approved by the UCLA Institutional Review Board (IRB#11-003190) and carried out wholly according to the Helsinki Declaration’s ethical standards.

### Protocol

**Figure 1:**
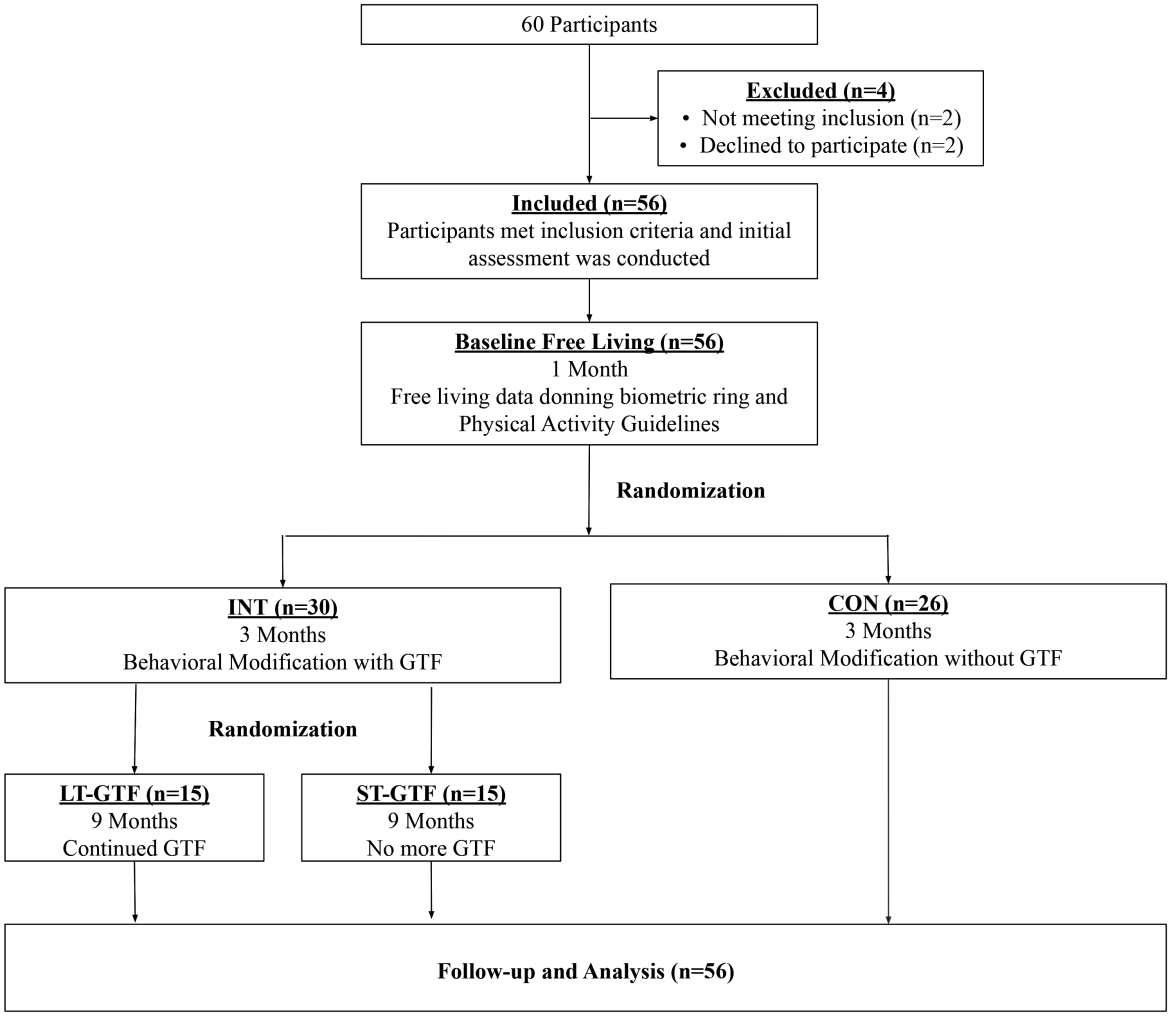
CONSORT diagram showing participant flow through the study. INT, Intervention; CON, Control; GTF, Guided Text Feedback; LT-GTF, Long-Term GTF; ST-GTF, Short-Term GTF.

This randomized study with concealed allocation and assessor blinding was conducted in the Airway & Exercise Physiology Research Laboratory at the David Geffen School of Medicine at UCLA. Participants were randomly allocated (1:1) to receive either a behavioral modification intervention with daily GTF (INT group) or a non-directive, equal attention wellness education placebo-controlled program (CON group) by an investigator independent of the recruitment of participants using an online-generated random number program. Allocation was concealed with the use of consecutively numbered envelopes. After 3months, those in the INT group were further randomized (1:1) to receive either daily GTF for the entire 12months (long-term GTF, LT-GTF group) or no further GTF (short-term GTF, ST-GTF group). A CONSORT flow diagram of the progression through the phases of the trail is shown in Figure 1. Both the behavioral modification intervention and wellness education program were conducted remotely *via* a smartphone application (UC Fit v1.11) downloaded to participants’ smartphones from either the Google Play Store or Apple App Store. Guided text message-based feedback was sent once per day (365 for the LT-GTF and 90 texts for the ST-GTF groups, respectively), using standard short message service (SMS). A trained research associate (in consultation with an experienced clinical sleep psychologist) provided GTF in addition to conducting the behavioral modification and equal attention sessions throughout the study.

#### Intervention Group: Behavioral Modification

The weekly behavioral modification intervention entailed twelve, 30-min digitally interactive whiteboard and screencasting presentations embedded into a smartphone app (UC Fit v1.11). Each presentation covered a different topic related to reducing stress, enhancing relaxation, or improving sleep, and provided individualized recommendations for implementation. The behavioral modification program was developed, successfully implemented, and published by the UC Fit lab during a previous study (Dolezal et al., 2019). It focused on altering the key mediators of behavior change, including self-efficacy, social support, and motivation. The content, commonly provided to individuals with induced insufficient sleep syndrome, was developed to encourage participants to implement stress management techniques, such as relaxation, problem-solving to adherence, and healthy sleep hygiene (Borbély, 1982; Morgenthaler et al., 2006; Miller and Rollnick, 2012).

#### Daily Guided Text Message-Based Feedback

During the course of the study, participants in ST-GTF and LT-GTF groups (combined as the INT group during months 0–3) received daily text message-based feedback that utilized social-cognitive behavioral models (Shaw et al., 2013; Collins et al., 2014; Fassnacht et al., 2015; Hall et al., 2015; Maddison et al., 2015; Yan et al., 2015) that incorporated a combination of positive reinforcement techniques. GTF was informed by a thorough review of the activity and sleep data from the prior 24h and personalized to each participant. Examples of GTF are illustrated in Table 1. Third-party access to each participant’s Oura app-enabled research associates to perform these reviews remotely and without additional disruption to the participant’s normal routine.

**Table 1:** Examples of guided text message-based feedback (GTF) for participant activity utilizing positive reinforcement techniques including informative, affirmatory, and persuasive content (i.e., guided).

Type	Text Message
Informative	You took 8,235 steps yesterday with 10% of time jogging
Affirmatory	Wonderful job! This week you increased in average step counts by 800 since last week
Persuasive	Your sleep and HRV trends this week indicate good sleep and adequate recovery. This week let us strive for a larger portion of your steps coming from jogging

#### Control Group: Wellness Education Program

To account for the non-specific effects of time and attention conferred by the intervention (independent of the sleep-related content), participants randomized to the CON group received equal time periods of education. Once per week, participants viewed a 30-min digital presentation within the UC Fit app that covered generalized healthy lifestyle advice and were absent of any specific guidance related to relaxation, stress reduction, or sleep. The wellness education program included the following topics: healthy relationships, brainpower, movement therapy (ergonomics), general health screening, environmental health, cancer screening, tobacco/nicotine, time management, basic hygiene practices, and preventable diseases/immunizations.

#### Exercise

The exercise intervention focused on increasing leisure-time physical activity – walking in particular – as well as encouragement to accrue incidental activity through daily tasks, such as household chores and active transport. All participants were instructed to walk or jog at least 150 to 300min per week in accordance with the American College of Sports Medicine Physical Activity (American College of Sports Medicine, 2017) and Center for Disease Control Physical Activity Guidelines (Centers for Disease Control and Prevention, 2018). Over time, participants receiving the intervention were encouraged to increase walking speeds to a comfortable jog.

### Baseline, Quarterly, and Post-test Outcome Measures

All outcomes were measured in-person at the lab or remotely using a multisensory wearable sleep and activity tracker. The primary outcome measure for this study was Sleep Quality measured by Sleep Onset Latency and Total Sleep time. Secondary outcomes included additional sleep metrics (listed below), Cardiovascular Fitness measured by VO_2_max, Physical Activity measured by total Step Count and % Time Jogging, Heart Rate Variability, and Body Composition.

#### In-Person: Anthropometric and Cardiovascular Fitness Laboratory Measures

All study participants visited the laboratory and were tested for anthropometrics (weight and percent body fat) and cardiovascular fitness. These assessments were conducted near the same time of day by the same examiner at baseline (0-month) and month 3 for CON group and at months 0, 3, 6, 9, and 12 for the INT group.

Height was determined using a precision stadiometer (Seca, Hanover, MD, United States), and body mass and percent body fat (BF%) were measured using a calibrated octipolar, multifrequency, multisegmental bioelectrical impedance analyzer scale (BIA; 270; InBody, Biospace, Cerritos, CA, United States). The BIA measurements were carried out according to the manufacturer’s instructions by a trained investigator. Briefly, each participant stood upright on the scale platform with the ball and heel of each foot on two metallic footpads while holding a handgrip with both hands pronated and perpendicular to the floor. The participant held the handgrip completely with the palm on one electrode and the thumb resting on the top of the unit’s other electrode. To ensure accuracy, participants adhered to standard pre-measurement BIA guidelines recommended by the American Society of Exercise Physiologists (Heyward, 2001).

A portable, lightweight (~800g) metabolic analyzer (PNOE, Palo Alto, CA), which has been previously validated (Tsekouras et al., 2019) and successfully utilized in exercise research (Browne et al., 2020; Robinson et al., 2020; Hu et al., 2021), was used to determine participants cardiovascular fitness (or aerobic capacity; VO_2_max) *via* an incremental, symptom-limited maximal treadmill exercise test. The PNOE was attached by a shoulder harness to the participants’ upper back while wearing a standard facemask and head support (Hans Rudolph, Inc., Shawnee, KS). The PNOE measured breath-by-breath ventilation as the participant breathed through a microelectromechanical hot film anemometer flow sensor that directed expired air to the gas analyzer for measures of oxygen consumption (VO_2_) and carbon dioxide production (VCO_2_). The gas analyzers were calibrated before each assessment per manufacturer instructions. Simultaneous time-aligned measures of heart rate were recorded *via* a chest strap (RS400; Polar Electro, Inc., Kempele, Finland).

#### Remote: Wearable Biometric (Sleep and Activity) Measures

**Figure 2:**
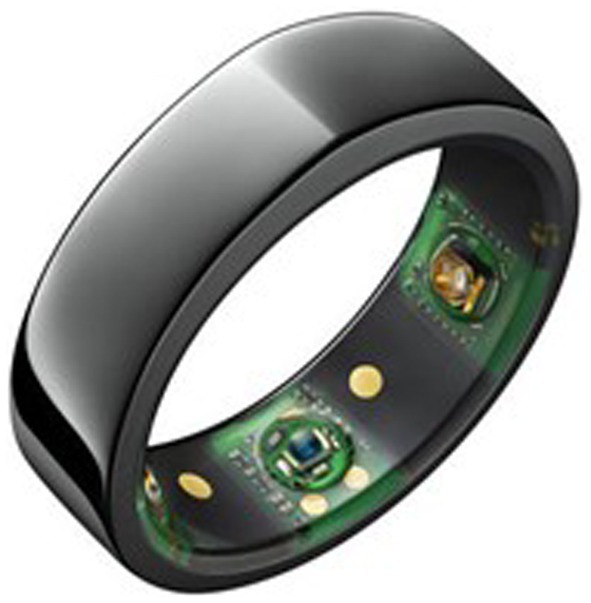
Commercially available multisensory wearable sleep and activity tracker (Oura ring 2nd generation; Oura Health, Oulu, Finland) highlighting sensor interface.

Prior to study initiation, each participant completed a detailed, step-by-step instructional tutorial on how to use the Oura ring (2nd generation), a commercially available multisensory wearable sleep and activity tracker (Ōura Health, Oulu, Finland). The biometric ring is shown in Figure 2. The OURA ring detects pulse rate, variation in inter-beat-intervals, and pulse amplitude from the finger optical pulse waveform (i.e., infrared photoplethysmography). The ring also measures motion *via* triaxial accelerometry (configured to record data at a sampling frequency of 50Hz and a resolution of ± 2g) and skin temperature that uses a negative temperature coefficient thermistor. The manufacture asserts to use these physiological signals (a combination of motion, nocturnal heart rate and heart rate variability, and pulse wave variability amplitude), in combination with sophisticated machine learning-based methods, to compute deep (PSG N3), light (PSG N1+N2), and rapid-eye-movement (REM) sleep as well as sleep/wake states (de Zambotti et al., 2019). Very recently, the Oura ring showed high accuracy wake-sleep detection, sleep staging sensitivity, and specificity that approaches results typically reported in PSG and EEG-based studies (Asgari Mehrabadi et al., 2020; Roberts et al., 2020).

For each participant, the finger (excluding pinky) demonstrating the best, snug fit for the waterproof, titanium-made ring was chosen. Oura rings come in different sizes (US standard ring sizes 6–13), weigh ~5g, and have a battery life of around 5–6 consecutive nights. The ring automatically connects *via* Bluetooth and transfers data to a mobile platform *via* the dedicated Oura application from either the Google Play Store or Apple App Store. Oura app notifications and firmware updates were disabled for the entirety of the study to control for potential confounding variables. Participants were asked to wear the ring 24/7 (except when charging) and to open the application every morning to automatically upload the data from the ring to the smartphone app. This ensured that both participants and research study associates were able to view the sleep, HRV, and activity data provided by the application.

Sleep metrics were defined as the following:

1. Bedtime (XX h XX min AM/PM): An estimate of the initial period intending to sleep.
2. Sleep Onset Latency (X h XX min): The time it takes for you to fall asleep.
3. Nocturnal Heart Rate (/min): Number of times your heart beats per minute while at rest.
4. Time in Bed (X h XX min): Number of hours between bedtime and wake-up time.
5. Total Sleep (X h XX min): Total amount of time spent in light, REM, and deep sleep.
6. Wake-up Time (XX hr. XX min AM/PM): Time one rises out of bed.

Heart rate variability (HRV) is utilized to reflect modulation in the autonomic nervous system (ANS). Typically, a low HRV value signifies increased sympathetic drive and ANS dysregulation, while a high HRV is indicative of greater parasympathetic activity and restoration of cardiovascular homeostasis (Taralov et al., 2015; Kim et al., 2018). HRV is also a practical biofeedback tool for improved relaxation and sleep, and an indicator of a person’s exercise recovery status and readiness to train (Plews et al., 2012; Hu et al., 2020). HRV has also been shown to be useful in predicting morbidities from common mental disorders (such as stress, depression, PTSD and anxiety) since these all increase sympathetic output and create a self-perpetuating cycle that produces autonomic balance and greater allostatic load (i.e., low HRV-rMSSD). In a recently validated study (Kinnunen et al., 2020), a high agreement between the Oura ring and gold-standard ECG was observed for nightly average HR and HRV (*r*^2^=0.996 and 0.980, respectively). Nocturnal HRV in this study was quantified *via* rMSSD (root mean square of successive R-R interval differences) as this is the HRV measurement collected and reported by the Oura Ring.

Activity metrics were defined as the following:

1. Step Count: Measurement of daily steps per 24h period
2. % Time Jogging: total steps >4.0 mph/total steps

### Statistical Analysis

Descriptive statistics are presented as mean±standard deviation (SD). All Oura ring measures were reported as 5-day rolling averages computed by the app. Statistical significance was determined based on *α*=0.05, and all tests were two-tailed. Continuous variables were first assessed for normality *via* Shapiro-Wilk tests. Homogeneity of variances and variances of the differences were assessed with Bartlett’s tests and Mauchly’s tests of sphericity, respectively. If Mauchly’s test of sphericity was violated, the Huynh-Feldt correction was utilized. Two-by-two and two-by-three mixed-model ANOVA were conducted. If significant main effects and/or interaction was found, pairwise comparisons using independent or paired t tests (for between-group and within-group comparisons, respectively) were then performed. In order to control the familywise error rate, a Bonferroni correction was employed. Effect sizes were measured by *η*^2^ following ANOVA and Hedges’ g following pairwise comparisons. Analysis was performed in Excel (Microsoft Corporation^®^, Redmond, Washington) and R (version 4.0.4; R Foundation for Statistical Computing^®^, Vienna, Austria).

## Results

For the first three months of the study, fifty-six adult volunteers ages 28–47years old (28 females) were enrolled and randomized to the control group (26 participants, 13 female) or intervention group (30 participants, 15 female; Table 2). Following the partitioning of the intervention group after month 3, 15 participants (7 female) in the LT-GTF group and 15 participants (8 female) in the ST-GTF group completed the entire 12-month study. Daily Oura ring data was successfully retrieved from 55 of the 56 study participants. One control participant had an unknown technical issue precluding any data from after the 3rd week. Thus, 99.44% (19,962) of the possible 20,075 daily Oura measures were successfully captured during the 12-month period. During the 30-day baseline collection prior to study start, there were eleven total days of missing data cases among the 56 participants: five due to initially low battery charge status prior to recording, and six short-term memory issues from not opening the associated Oura App for data download within two days (memory limitation).

**Table 2:** Demographics at baseline for all participants in randomized groups.

	Control (*n* =26)	LT-GTF (*n* =15)	ST-GTF (*n* =15)
Sex (M, F)	*n* =13, *n* =13	*n* =8, *n* =7	*n* =7, *n* =8
Age (yr)	35.7±6.3	36.3±6.1	35.6±5.6
Height (cm)	172.7±7.1	171.45±4.3	173.5±5.8
Body mass (kg)	73.9±10.1	74.1±10.5	73.5±9.2
Body fat (%)	27.4±2.2	27.2±2.4	26.3±2.2
VO2 Max (ml/kg/min)	35.0±0.6	36.4±1.4	36.3±0.7
HRV-rMSSD (ms)	36.7±2.5	30.3±1.7	30.1±1.8

*Values are mean±SD. GTF, Guided Text Feedback; LT-GTF, Long-Term GTF; ST-GTF, Short-Term GTF*.

### Anthropometrics

**Figure 3:**
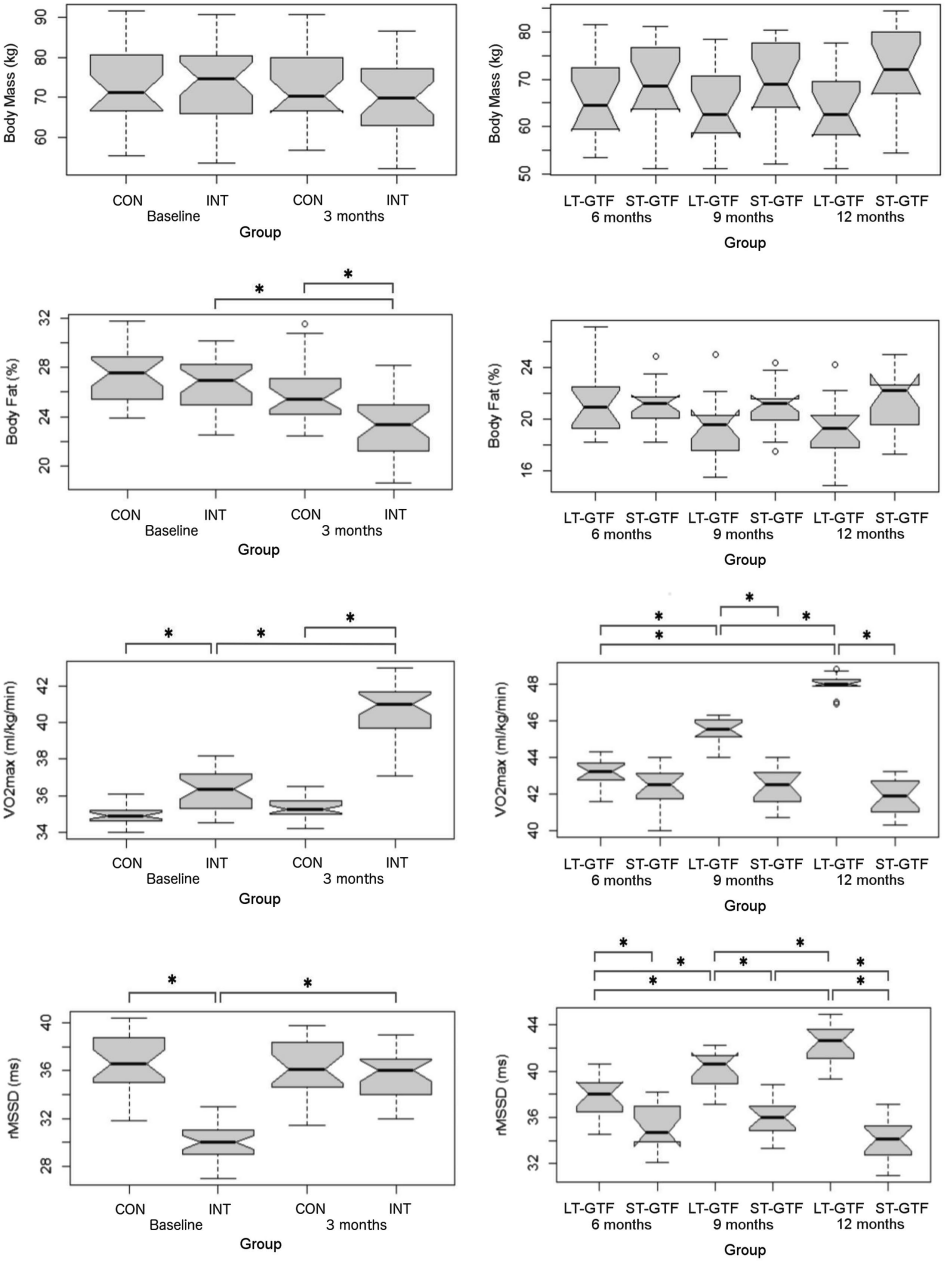
Anthropometrics (Body Mass, BF%), cardiovascular fitness (VO_2_max), and autonomous nervous system (rMSSD) data. Graphs on the left represent control (CON) and intervention (INT) groups from baseline to month 3. Graphs on the right represent measurements taken at 6, 9, and 12months. LT-GTF, Long-term GTF group; ST-GTF, Short-term GTF. ^*^Represent statistically significant differences.

There was no significant difference in age or height across groups. Participants’ characteristics are included in Table 2. From 0–3 months and 6–12months, there was a significant main effect of time (*p*<0.001; *η*^2^=0.01) as well as an interaction between group and time (*p*<0.001; *η*^2^=0.01) on body mass; however, pairwise comparisons did not reveal any significant differences between groups or time points (Figure 3).

Regarding BF%, there were significant main effects of group (*p*<0.001; *η*^2^=0.13) and time (*p*<0.001; *η*^2^=0.24), as well as an interaction between group and time (*p*=0.006; *η*^2^=0.06) from 0–3 months (Figure 3). Pairwise comparisons demonstrated a significant reduction in BF% from 26.8±2.3 to 23.0±2.5% (*p*<0.001; *g*=1.57) in the intervention group. After 3months, the intervention group recorded a significantly lower BF% compared to the control group (23.0±2.5 vs. 25.9±2.4%; *p*<0.001; *g*=1.17). From 4–12 months, there was a significant main effect of time (*p*<0.001; *η*^2^=0.05) as well as an interaction between group and time (*p*<0.001; *η*^2^=0.06); however, pairwise comparisons did not reveal any significant differences between groups or time points (Figure 3).

### Sleep Metrics

**Figure 4:**
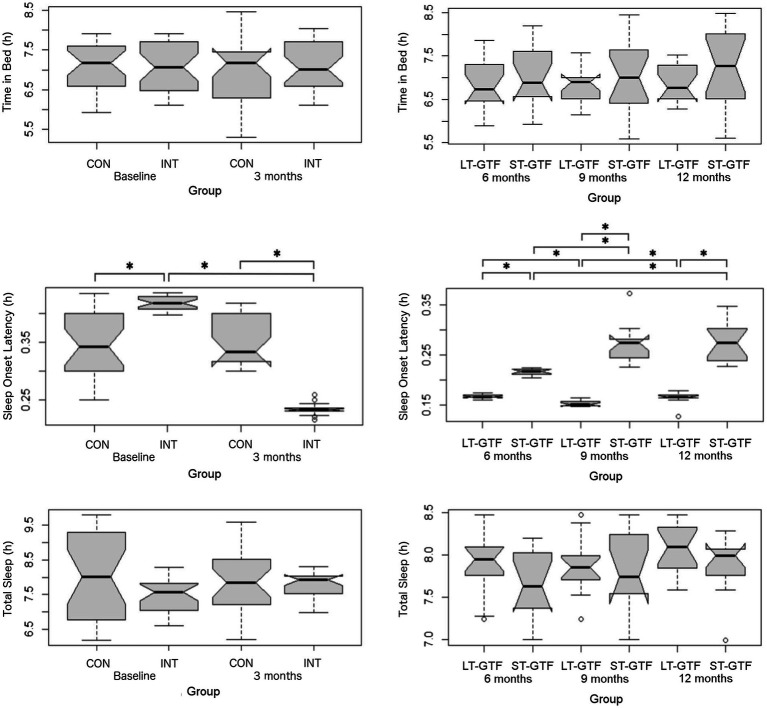
Sleep metrics (Time in Bed, Sleep Onset Latency, and Total Sleep). Graphs on the left represent control (CON) and intervention (INT) groups from baseline to month 3. Graphs on the right represent measurements taken at 6, 9, and 12months. LT-GTF, Long-term GTF group; ST-GTF, Short-term GTF. ^*^Represent statistically significant differences.

There was no significant difference regarding Time in Bed between groups or time points (Figure 4). Regarding sleep onset latency (SOL), there were significant main effects of group (*p*=0.002; *η*^2^=0.10) and time (*p*<0.001; *η*^2^=0.63), as well as an interaction between group and time (*p*<0.001; *η*^2^=0.67) from 0–3 months (Figure 4). Pairwise comparisons showed a decrease from 0.42±0.01 to 0.23±0.01h (*p*<0.001; *g*=18.00) in the intervention group. At baseline (0months), the intervention group demonstrated a longer SOL than the control group (0.42±0.01 vs. 0.34±0.06h; *p*<0.001; *g*=1.83). However, after 3months, the intervention group demonstrated a shorter SOL than the control group (0.23±0.01 vs. 0.35±0.04h; *p*<0.001; *g*=4.39).

From 4–12 months (Figure 4), there were significant main effects of group (*p*<0.001; *η*^2^=0.82) and time (*p*<0.001; *η*^2^=0.24), as well as an interaction between group and time (*p*<0.001; *η*^2^=0.34). Within the LT-GTF group, pairwise comparisons revealed a decrease in SOL from month 6 to month 9 (0.17±0.00 vs. 0.15±0.01h; *p*<0.001; *g*=3.15) but an increase from month 9 to month 12 (0.15±0.01 vs. 0.17±0.01h; *p*=0.002; *g*=1.38). Within the ST-GTF group, pairwise comparisons demonstrated an increase in SOL from month 6 to month 9 (0.22±0.01 vs. 0.27±0.04h; *p*<0.001; *g*=2.20) and from month 6 to month 12 (0.22±0.01 vs. 0.28±0.04h; *p*<0.001; *g*=2.13). After 6months, the LT-GTF group showed a shorter SOL compared to the ST-GTF group (0.17±0.00 vs. 0.22±0.01h; *p*<0.001; *g*=8.03). Similarly, after 9months, the LT-GTF group showed a shorter SOL compared to the ST-GTF group (0.15±0.01 vs. 0.27±0.04h; *p*<0.001; *g*=4.77). Likewise, after 12months, the LT-GTF group also demonstrated a shorter SOL than the control group (0.17±0.01 vs. 0.28±0.04h; *p*<0.001; *g*=3.75).

Regarding Total Sleep, there were no significant main effects or an interaction from 0 to 3 months. From 4 to 12 months, there was a significant main effect (*p*<0.001; *η*^2^=0.05) of time, but not group, nor an interaction. Follow-up pairwise comparisons examining the different time points did not reveal any significant differences (Figure 4).

### Physical Activity

**Figure 5:**
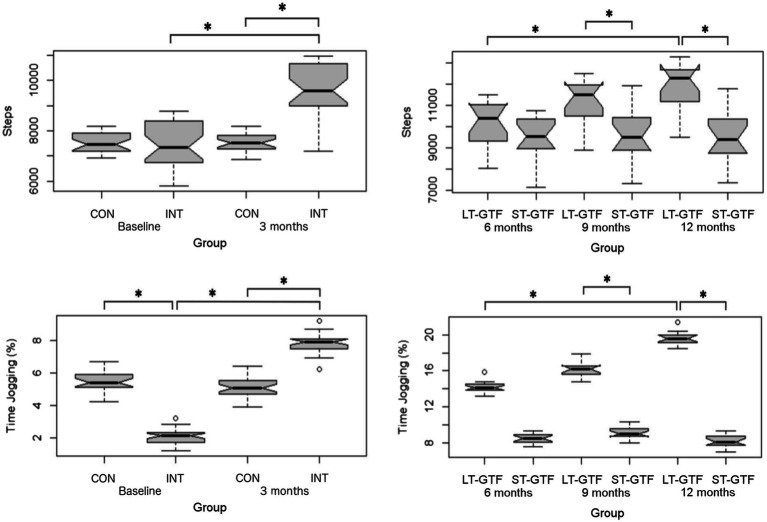
Physical activity measurements (Step Count, % Time Jogging). Graphs on the left represent control (CON) and intervention (INT) groups from baseline to month 3. Graphs on the right represent measurements taken at 6, 9, and 12months. LT-GTF, Long-term GTF group; ST-GTF, Short-term GTF. ^*^Represent statistically significant differences.

Regarding Step Count, there were significant main effects of group (*p*<0.001; *η*^2^=0.32) and time (*p*<0.001; *η*^2^=0.35), as well as an interaction between group and time (*p*<0.001; *η*^2^=0.35) from 0–3months. Pairwise comparisons showed an increase from 7,446±907 to 9,626±998 steps (*p*<0.001; *g*=2.26) in the intervention group. After 3months (Figure 5), the intervention group demonstrated a greater Step Count than the control group (9,626±998 vs. 7,527±359 steps; *p*<0.001; *g*=2.68).

From 4 to 12months (Figure 5), there were significant main effects of group (*p*<0.001; *η*^2^=0.34) and time (*p*<0.001; *η*^2^=0.11), as well as an interaction between group and time (*p*<0.001; *η*^2^=0.10). Within the LT-GTF group, pairwise comparisons revealed an increase in Step Count from month 6 to month 12 (10,126±1,073 vs. 11,920±1,073 steps; *p*<0.001; *g*=1.63). After 9months, the LT-GTF group showed a greater Step Count compared to the ST-GTF group (11,171±1,034 vs. 9,581±1,160 steps; *p*<0.001; *g*=1.41). Similarly, after 12months, the LT-GTF group also demonstrated a greater Step Count than the control group (11,920±1,073 vs. 9,527±1,153 steps; *p*<0.001; *g*=2.09).

Regarding % Time Jogging, there were significant main effects of group (*p*=0.03; *η*^2^=0.07) and time (*p*<0.001; *η*^2^=0.87), as well as an interaction between group and time (*p*<0.001; *η*^2^=0.89) from 0–3 months (Figure 5). Pairwise comparisons showed an increase from 2.1±0.4 to 7.8±0.6% (*p*<0.001; *g*=11.29) in the intervention group. At baseline (0months), the intervention group demonstrated a smaller % Time Jogging than the control group (2.1±0.4 vs. 5.4±0.6%; *p*<0.001; *g*=6.54). However, after 3months, the intervention group demonstrated a greater % Time Jogging than the control group (7.8±0.6 vs. 5.1±0.6%; *p*<0.001; *g*=4.80).

From 4–12 months (Figure 5), there were significant main effects of group (*p*<0.001; *η*^2^=0.97) and time (*p*<0.001; *η*^2^=0.70), as well as an interaction between group and time (*p*<0.001; *η*^2^=0.77). Within the LT-GTF group, pairwise comparisons revealed an increase in %time jogging from month 6 to month 12 (14.2±0.7 vs. 19.6±0.7%; *p*<0.001; *g*=7.41). After 9months, the LT-GTF group showed a greater %time jogging compared to the ST-GTF group (16.1±0.8 vs. 9.1±0.7%; *p*<0.001; *g*=9.24). Similarly, after 12months, the LT-GTF group also demonstrated a greater % Time Jogging than the control group (19.6±0.7 vs. 8.2±0.7%; *p*<0.001; *g*=14.97).

### Cardiovascular Fitness

Regarding VO_2_max, there were significant main effects of group (*p*<0.001; *η*^2^=0.71) and time (*p*<0.001; *η*^2^=0.54), as well as an interaction between group and time (*p*<0.001; *η*^2^=0.45) from 0–3 months (Figure 3). Pairwise comparisons showed an increase from 36.3±1.1 to 40.6±1.6ml/kg/min (*p*<0.001; *g*=3.04) in the intervention group. At baseline (0months), the intervention group demonstrated a greater VO_2_max than the control group (36.3±1.1 vs. 35.0±0.6ml/kg/min; *p*<0.001; *g*=1.57). A similar finding occurred after 3months: the intervention group demonstrated a greater VO_2_max than the control group (40.6±1.6 vs. 35.4±0.6ml/kg/min; *p*<0.001; *g*=4.14).

From 4–12 months (Figure 3), there were significant main effects of group (*p*<0.001; *η*^2^=0.78) and time (p<0.001; *η*^2^=0.52), as well as an interaction between group and time (p<0.001; *η*^2^=0.62). Within the LT-GTF group, pairwise comparisons revealed increases in VO_2_max from month 6 to month 9 (43.1±0.8 vs. 45.5±0.7ml/kg/min; *p*<0.001; *g*=3.04), from month 6 to month 12 (43.1±0.8 vs. 48.0±0.5ml/kg/min; *p*<0.001; *g*=6.95), and from month 9 to month 12 (45.5±0.7 vs. 48.0±0.5ml/kg/min; *p*<0.001; *g*=4.09). After 9months, the LT-GTF group showed a greater VO_2_max compared to the ST-GTF group (45.5±0.7 vs. 42.5±1.1ml/kg/min; *p*<0.001; *g*=3.28). A similar finding occurred after 12months: The LT-GTF group demonstrated a greater VO_2_max than the control group (48.0±0.5 vs. 41.9±1.0ml/kg/min; *p*<0.001; *g*=7.43).

### Heart Rate Variability

Regarding HRV, there were significant main effects of group (*p*<0.001; *η*^2^=0.41) and time (p<0.001; *η*^2^=0.24), as well as an interaction between group and time (*p* <0.001; *η*^2^=0.31) from 0–3months (Figure 3). Pairwise comparisons showed an increase from 30.2±1.7 to 35.5±1.9ms (*p*<0.001; *g*=2.89) in the intervention group. At baseline (0months), the intervention group demonstrated a smaller rMSSD than the control group (30.2±1.7 vs. 36.7±2.5ms; *p*<0.001; *g*=3.05).

From 4–12months (Figure 3), there were significant main effects of group (*p*<0.001; *η*^2^=0.65) and time (*p*<0.001; *η*^2^=0.16), as well as an interaction between group and time (*p*<0.001; *η*^2^=0.33). Within the LT-GTF group, pairwise comparisons revealed increases in rMSSD from month 6 to month 9 (37.7±1.9 vs. 40.0±1.7ms; *p*=0.001; *g*=1.28), from month 6 to month 12 (37.7±1.9 vs. 42.3±1.8ms; *p*<0.001; *g*=2.44), and from month 9 to month 12 (40.0±1.7 vs. 42.3±1.8ms; *p*=0.002; *g*=1.24). Within the ST-GTF group, pairwise comparisons demonstrated a decrease in rMSSD from month 9 to month 12 (36.1±1.7 vs. 34.1±1.7ms; *p*=0.004; *g*=1.11). After 6months, the LT-GTF group showed a greater rMSSD compared to the ST-GTF group (37.7±1.9 vs. 35.2±1.9ms; p=0.002; *g*=1.20). Similarly, after 9months, the LT-GTF group showed a greater rMSSD compared to the ST-GTF group (40.0±1.7 vs. 36.1±1.7ms; *p*<0.001; *g*=2.24). Likewise, after 12months, the LT-GTF group also demonstrated a greater rMSSD than the control group (42.3±1.8 vs. 34.1±1.7ms; *p*<0.001; *g*=4.52).

## Discussion

**Figure 6:**
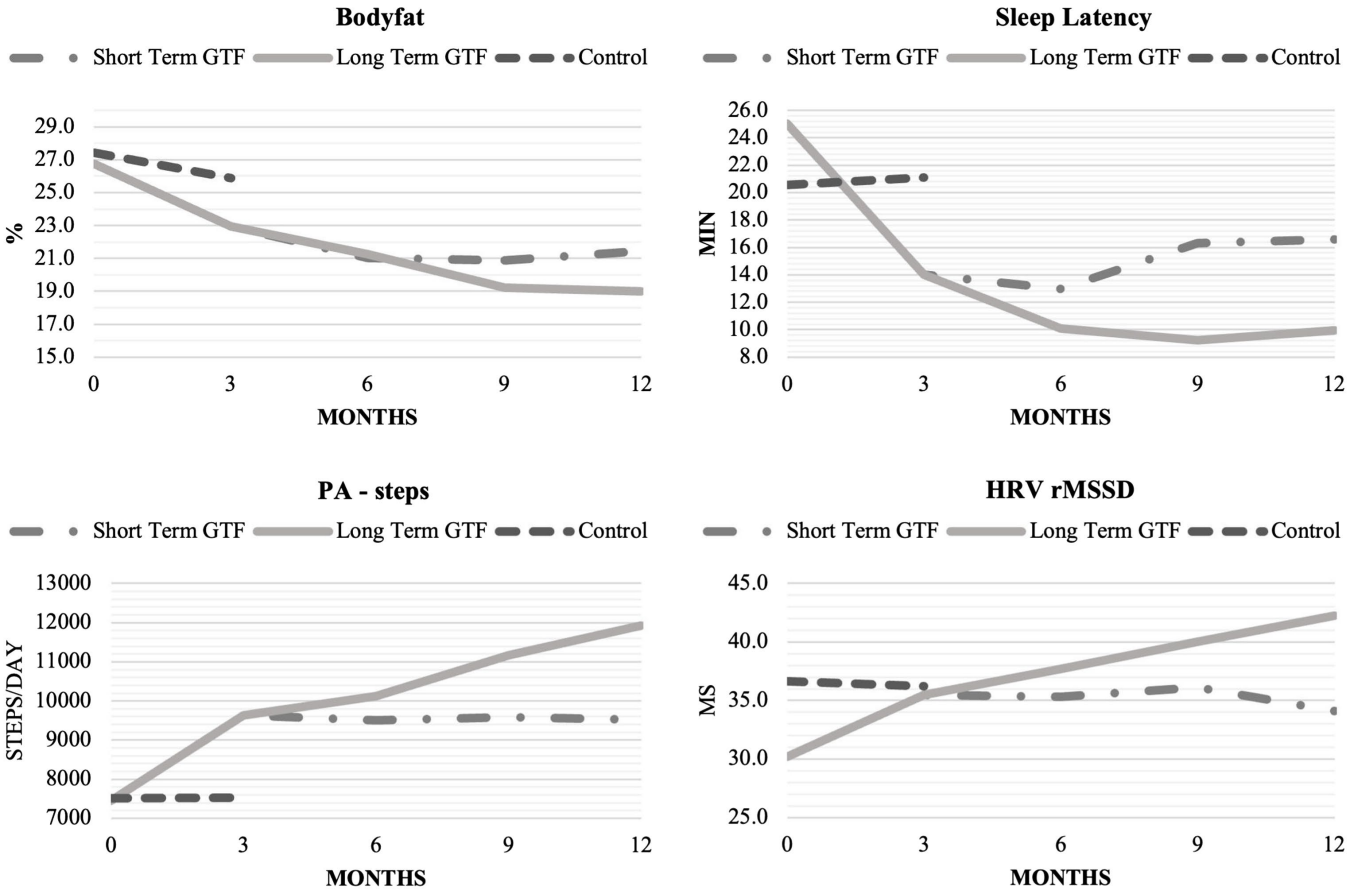
Evolution of Body Fat %, Sleep Onset Latency, Step Count, and rMSSD during the study.

This study demonstrates the efficacy of utilizing wearable technology in remote delivery of an app-based intervention to improve sleep, activity levels, fitness, and health. In general, a trend emerged across several key outcome measures, affirming the importance of including guidance and encouragement, provided in this study through GTF, as key components of a successful behavior modification intervention. The CON group showed no difference in SOL, Step Count, % Time Jogging, VO_2_max, BF%, and HRV, while significant improvement occurred among the INT group over the first three months. During the following nine months, those who continued to receive GTF (LT-GTF group) continued to improve in each of these outcomes except BF%, while scores for those who received no further GTF (ST-GTF group) remained relatively stagnant or regressed slightly. Figure 6 illustrated trends for SOL, Step Count, BF%, and HRV over the course of the study.

SOL is commonly used to aid in the diagnosis of insomnia and to monitor the efficacy of treatment, yet there is no widespread agreement on a clinical threshold. Many researchers and clinicians employ a diagnostic cutoff of >30min (Martin and Ancoli-Israel, 2002; Lichstein et al., 2003); however, Lineberger et al. (Lineberger et al., 2006) demonstrated that a cutoff value of >20min best-discriminated individuals with insomnia from normal sleepers. This threshold has been endorsed in more recent publications (Edinger, 2016; Exelmans et al., 2018). With baseline latencies of 20.6min for the CON group and 25.0min for the INT group, participants in the current study could be considered borderline to meet these criteria for insomnia. Thus, it is meaningful that SOL in the INT group improved to 14.0min at the 3-month follow-up, dropping below the 20-min insomnia threshold and exceeding the published criteria of 10-min improvements for clinical importance (Sateia et al., 2017). In fact, with SOL improvements of −11min at 3months and−15min at 12months among the LT-GTF group, the GTF-enabled behavioral intervention rivaled or outperformed 13 of 14 commonly used pharmacologic treatments analyzed in a recent systematic review and Clinical Practice Guideline commissioned by the American Academy of Sleep Medicine (Sateia et al., 2017). Many considerations factor into treatment decisions for insomnia. Still, these results indicate that behavioral interventions similar to the one presented in this study should be considered, especially among those with mild or sub-clinical insomnia or for whom the pharmacologic intervention is contraindicated.

The continued improvement in SOL among the LT-GTF group is noteworthy. Most interesting, however, is the sustained healthy values of the ST-GTF group, which received no further external engagement after the short-term 3-month intervention, with both groups remaining under the 20-min insomnia threshold at the 12-month follow-up. A similar sustained effect was found in a recent study by Nguyen et al., which demonstrated maintained improvement in sleep disturbances and PSQI score 12weeks after stopping the behavioral feedback and health coaching intervention. Unlike the present study, however, subjects continued using the wearable fitness and sleep tracker (Nguyen et al., 2021). Similar to the present, Nguyen et al. included a control group that wore a fitness tracker without feedback for a three-month period. Although the control group recorded small improvements in wake after sleep onset (WASO) and sleep efficiency, only the feedback group improved number of awakenings (NWAKE) and Pittsburgh sleep quality index (PSQI) score. Taken together, these studies suggest that simply wearing a fitness tracker in isolation is insufficient to realize a meaningful change in most people. Perhaps more importantly, these studies also establish that a relatively brief period of guided feedback may be enough to enact long-term change. While continued feedback appears to produce continued improvement, a short-term period of personalized coaching at the outset of using a fitness tracker may be sufficient to achieve meaningful results and create long-lasting, healthy habits.

This pattern of initial and continued improvement with GTF, and maintenance of improved values upon removal of GTF, was also observed in Step Count, % Time Jogging, and VO_2_max. Previous research demonstrates similar improvements in physical activity and cardiovascular fitness resulting from interventions incorporating activity trackers and personalized feedback (Braakhuis et al., 2019; Brickwood et al., 2019, 2021; Ellingson et al., 2019). Furthermore, there is a dose-response relationship between physical activity and major health outcomes, where simply increasing daily step count has a positive impact on mortality and morbidity risk. A widely reported problem with physical activity interventions is low compliance in maintaining the new lifestyle (van der Bij et al., 2002; Müller-Riemenschneider et al., 2008); however, better long-term adaptations have been shown when interventions incorporate feedback and personalized behavioral change techniques compared to generic activity recommendations or activity trackers alone (Dolezal et al., 2015; Ellingson et al., 2019; Brickwood et al., 2021). A study by Ellingson et al. recruited participants with a wide range of physical activity levels and found that among those entering their study with higher activity levels, combining activity tracker use with personalized coaching techniques such as motivational interviewing and habit education was more effective at maintaining high activity levels than receiving an activity tracker alone (Ellingson et al., 2019). This reinforces the previously discussed potential for short-term feedback to facilitate long-term changes.

As our intervention targeted multiple lifestyle factors simultaneously, it is possible the effects observed in certain outcome measures are at least partially the result of improvements made in other related outcomes. Indeed, there is ample evidence indicating that exercise positively affects sleep quality (Yang et al., 2012), and since we did not include groups receiving only partial intervention (e.g., sleep coaching, but no physical activity encouragement), we are unable to conclusively determine to what extent observed improvements in SOL were due solely to an effective sleep intervention. However, prior research has demonstrated that the effects of exercise on sleep are highly variable depending on the age of the participant, and the intensity, and timing of exercise sessions. Additionally, not all sleep-related outcomes are equally affected by exercise (Dolezal et al., 2017). A recent systematic review by Dolezal et al. found good evidence that exercise promoted increased sleep efficiency and duration, yet 8 of 13 studies reporting SOL found no change resulting from exercise (Dolezal et al., 2017). Similarly, a meta-analysis by Banno et al. (2018) found benefits from exercise in subjective measures, such as the PSQI and insomnia severity index (ISI), but found no effect on directly measured sleep latency. Based on these findings, we believe it reasonable to state that improvements in SOL observed in the current study are evidence of an effective sleep intervention, although parsing this out further in future studies involving wearable technology and remotely delivered interventions would be warranted. Regardless, the observed improvement in the current study underscores the importance of a whole-person and whole-lifestyle approach to improved health.

Measuring rMSSD HRV provided a valuable measure of parasympathetic activity and cardiovascular homeostasis restoration. This outcome measure followed a similar pattern to the others discussed, with continued improvement observed in the LT-GTF group and no meaningful change for better or worse during periods when GTF was withheld. To our knowledge, there is no reported minimal clinically important change (MCID) for HRV metrics; however, a recent study reported Minimal Detectable Change scores between 6.92–9.81ms for rMSSD (Bassi et al., 2018), indicating the 12.1ms improvement observed among the LT-GTF group at 12months may be noteworthy. Multiple studies have shown that physical activity positively modulates HRV (Dixon et al., 1992; Malfatto et al., 1996; Sandercock et al., 2007), and HRV may positively influence sleep (Zhuang et al., 2005). Higher HF HRV metrics found during a pre-bedtime resting period have been associated with shorter sleep latency and fewer arousal events (Werner et al., 2015), while another study found higher rMSSD values to be predictive of shorter WASO times (Fantozzi et al., 2019). Therefore, the results of the current study support previous research suggesting a positive relationship between increased HRV and improved sleep metrics.

Wearables have the potential to be at the forefront of preventative medicine by allowing patients to take control of their own healthcare and well-being. For many people, it may be difficult to gauge one’s activity level and quality of sleep (Ferrari et al., 2007; Lauderdale et al., 2008). Providing users with regular biometric feedback eliminates subjectivity and allows the user to obtain a more accurate representation of their current health status. The improvements seen in SOL, Step Count, % Time Jogging, VO2max, BF%, and HR show wearables to be a promising means to generate lasting positive impacts on users’ health and overall quality of life.

### Applications

Wearables are a relatively inexpensive yet valid, reliable, and feasible way to support health-related behavior change and a more active lifestyle (Kinnunen et al., 2019; Lynch et al., 2019). Electronic Health interventions have been shown to produce significant weight loss for individuals through applied self-monitoring, feedback, goal setting, shaping knowledge, and social support (Asbjørnsen et al., 2019). Previous research has also suggested that wearable devices’ promotion of physical activity and weight loss, combined with the ability to remotely capture and report real-life data, may be useful for health professionals with patient monitoring and support. Thus, there is growing interest from academic researchers and clinicians to better understand how to optimize this technology (Ringeval et al., 2020; Altini and Kinnunen, 2021). By leveraging wearable technology, the easy accessibility of personal health data has the potential to elicit significant behavioral changes when combined with personalized feedback to promote a more active and healthier lifestyle. Thus, contributing to increased healthspan and lifespan.

### Limitations

The interventional group consisted of a small sample size for the LT-GTF (*n*=15) and ST-GTF (*n*=15) groups. This limitation is likely mitigated by the study’s 1-month collection of baseline data, 12-month study duration, and strong participant adherence to wearing the biometric ring; however, future studies may consider larger sample sizes to generate greater external validity. Additionally, due to the lack of long-term studies conducted on the efficacy of wearables for sustained lifestyle modification, we have little to compare the efficacy of the sleep and activity interventions from other wearable devices that may offer additional benefits and behavior-modifying features. Future research is warranted considering the increasing digitalization of health, user accessibility of fitness wearables and suggested benefits indicated in this study.

## Conclusion

The utilization of biometric trackers has become increasingly widespread in our data-driven society. By providing data regarding physical activity, sleep, and other fitness metrics, research has previously shown wearables have the potential to promote improved health and lifestyle. However, despite the extensive data available from a fitness tracker, many users do not consistently comply with or utilize the advantages of fitness tracking in the long term to promote healthy lifestyle habits. Moreover, visually appealing graphs from “big” data misses the point; that is, wearable data should be used as a medium that empowers positive changes in users lives not merely as a collection device with the expectation the user finds it interesting and actionable. That rarely happens. This study underscores the importance of integrating these devices in tailored, personalized behavioral interventions that provide dynamic educational content, real-time encouragement and feedback with progressive goal setting. In the present study, this combination resulted in improved sleep quality and increased physical activity and fitness. Perhaps most notably, our data show that improvements achieved from a short duration of personalized coaching are largely maintained with the continued use of a wearable biometric device.

## Data Availability Statement

The original contributions presented in the study are included in the article/supplementary material, and further inquiries can be directed to the corresponding author.

## Ethics Statement

The studies involving human participants were reviewed and approved by UCLA Institutional Review Board. The patients/participants provided their written informed consent to participate in this study.

## Author Contributions

JB and BD conceived and coordinated the study and wrote the first manuscript draft. DB, JB, and EN participated in the conception of the study, writing of the first manuscript draft, and overall coordination of the study. BD and EN contributed to data collection and analysis. PG contributed to data collection. KI, QH, DG, and MP contributed to the writing of all subsequent manuscript drafts and a review of the literature. All authors read and provided significant input to all manuscript drafts, agreed to be accountable for all aspects of the work, and approved the final manuscript draft for publication.

## Conflict of Interest

The authors declare that the research was conducted in the absence of any commercial or financial relationships that could be construed as a potential conflict of interest.

## Publisher’s Note

All claims expressed in this article are solely those of the authors and do not necessarily represent those of their affiliated organizations, or those of the publisher, the editors and the reviewers. Any product that may be evaluated in this article, or claim that may be made by its manufacturer, is not guaranteed or endorsed by the publisher.
